# Synergistic Activity of Repurposed Peptide Drug Glatiramer Acetate with Tobramycin against Cystic Fibrosis Pseudomonas aeruginosa

**DOI:** 10.1128/spectrum.00813-22

**Published:** 2022-06-21

**Authors:** Ronan A. Murphy, Matthew Coates, Sophia Thrane, Akshay Sabnis, James Harrison, Silke Schelenz, Andrew M. Edwards, Thomas Vorup-Jensen, Jane C. Davies

**Affiliations:** a National Heart and Lung Institute, Imperial College London, London, United Kingdom; b Department of Biomedicine, Aarhus University, Aarhus, Denmark; c MRC Centre for Molecular Bacteriology and Infection, Imperial College London, London, United Kingdom; d Cycle Pharmaceutical Ltd., Cambridge, United Kingdom; e King’s College Hospital NHS Foundation Trust, KingsPath Clinical Diagnostics Pathology Services, London, United Kingdom; f Department of Paediatric Respiratory Medicine, Royal Brompton Hospital, London, United Kingdom; Louis Stokes Cleveland VAMC

**Keywords:** *Pseudomonas aeruginosa*, antibiotic resistance, antibiotic resistance breaker, antimicrobial peptides, cystic fibrosis, drug repurposing, synergism

## Abstract

Pseudomonas aeruginosa is the most common pathogen infecting the lungs of people with cystic fibrosis (CF), causing both acute and chronic infections. Intrinsic and acquired antibiotic resistance, coupled with the physical barriers resulting from desiccated CF sputum, allow P. aeruginosa to colonize and persist in spite of antibiotic treatment. As well as the specific difficulties in eradicating P. aeruginosa from CF lungs, P. aeruginosa is also subject to the wider, global issue of antimicrobial resistance. Glatiramer acetate (GA) is a peptide drug, used in the treatment of multiple sclerosis (MS), which has been shown to have moderate antipseudomonal activity. Other antimicrobial peptides (AMPs) have been shown to be antibiotic resistance breakers, potentiating the activities of antibiotics when given in combination, restoring and/or enhancing antibiotic efficacy. Growth, viability, MIC determinations, and synergy analysis showed that GA improved the efficacy of tobramycin (TOB) against reference strains of P. aeruginosa, reducing TOB MICs and synergizing with the aminoglycoside. This was also the case for clinical strains from people with CF. GA significantly reduced the MIC_50_ of TOB for viable cells from 1.69 mg/L (95% confidence interval [CI], 0.26 to 8.97) to 0.62 mg/L (95% CI, 0.15 to 3.94; *P* = 0.002) and the MIC_90_ for viable cells from 7.00 mg/L (95% CI, 1.18 to 26.50) to 2.20 mg/L (95% CI, 0.99 to 15.03; *P* = 0.001), compared to results with TOB only. Investigation of mechanisms of GA activity showed that GA resulted in significant disruption of outer membranes, depolarization of cytoplasmic membranes, and permeabilization of P. aeruginosa and was the only agent tested (including cationic AMPs) to significantly affect all three mechanisms.

**IMPORTANCE** The antimicrobial resistance crisis urgently requires solutions to the lost efficacy of antibiotics. The repurposing of drugs already in clinical use, with strong safety profiles, as antibiotic adjuvants to restore the efficacy of antibiotics is an important avenue to alleviating the resistance crisis. This research shows that a clinically used drug from outside infection treatment, glatiramer acetate, reduces the concentration of tobramycin required to be effective in treating Pseudomonas aeruginosa, based on analyses of both reference and clinical respiratory isolates from people with cystic fibrosis. The two agents acted synergistically against P. aeruginosa, being more effective combined *in vitro* than predicted for their combination. As a peptide drug, glatiramer acetate functions similarly to many antimicrobial peptides, interacting with and disrupting the P. aeruginosa cell wall and permeabilizing bacterial cells, thereby allowing tobramycin to work. Our findings demonstrate that glatiramer acetate is a strong candidate for repurposing as an antibiotic resistance breaker of pathogenic P. aeruginosa.

## INTRODUCTION

Pseudomonas aeruginosa is a Gram-negative, rod-shaped bacterium found ubiquitously in the environment and frequently associated with opportunistic infections (burns, wounds, eye infections). P. aeruginosa is the most common infecting bacteria in the lungs of people with cystic fibrosis (CF) (1). Cystic fibrosis is a genetic, life-limiting disorder and while it is a multisystem illness, lung disease causes the majority of morbidity and mortality in people with CF; impaired mucociliary clearance leads to chronic bacterial infection, significant inflammation, and bronchiectasis, resulting ultimately in respiratory failure (2–4). Forty-one percent of adult CF patients (>16 years of age) in the United Kingdom are chronically infected with P. aeruginosa, with a peak of 54% in the age 36 to 39 years cohort (1). P. aeruginosa is of particular concern due its ability to evade antibiotic treatments (via both innate and acquired mechanisms), leading to its designation as an ESKAPE pathogen by the World Health Organization (5–8).

The aminoglycoside tobramycin is one of the most commonly used antipseudomonal antibiotics in cystic fibrosis. Administration is either intravenous (i.v.) or inhaled directly to the airway (1). Limitations of the i.v. route include a narrow therapeutic index requiring concentration monitoring to avoid oto- and nephrotoxicity. The inhaled route allows high local levels with less systemic exposure, but once infection is chronic, drug efficacy is modest and eradication rare, a limitation shared with other agents at this disease stage. In part, this may relate to heterogeneity of deposition within partially obstructed airways, giving rise to subtherapeutic drug concentrations. Few new treatments are being developed for chronic P. aeruginosa, and the growing adult population means this unmet need will persist into the era of novel modulator drugs targeting the underlying cellular defect in CF (9–12).

A potential solution to the need for novel P. aeruginosa therapy, among the dearth of new antibacterial treatments, is the deployment of antimicrobial peptides (AMPs), which are small biological molecules, usually consisting of 10 to 50 amino acid residues, that form part of innate immune systems produced widely across all kingdoms of life (13–15). They frequently function via interactions with the bacterial membrane, which lead to cell wall weakening, thinning and/or permeabilization, and rapid cell death (13, 16–18). The Gram-negative membrane consists of an outer membrane, containing outwards-protruding lipopolysaccharide (LPS), and a cytoplasmic membrane, with a thin peptidoglycan layer between the two. AMPs are of interest not only because of their own potential antimicrobial activity but also for their possible use as antibiotic adjuvants; given in combination with antibiotics, they may reduce concentrations of the latter required for antimicrobial activity (19). This property, often referred to as “antibiotic resistance breaking,” has been reported against P. aeruginosa: the human-derived AMP LL-37 and its derivatives with azithromycin, vancomycin (20, 21), and novel synthetic peptides with tobramycin and colistin (22–24). However, despite promising results, many AMPs have significant barriers to clinical use, including concerns around cytotoxic effects (16, 25, 26).

Glatiramer acetate (GA) is an immunomodulator drug currently in clinical use in the treatment of multiple sclerosis (MS) (27–29). It is produced by the random polymerization of the four N-carboxy-α-amino acid anhydrides of l-glutamate, l-lysine, l-alanine, and l-tyrosine. The resulting peptidic copolymers biochemically resemble AMPs. We showed previously that GA has moderate antimicrobial activity that is comparable to that of LL-37 against P. aeruginosa: for both reference strains and clinical strains from people with CF, optimal activity occurred at 50 mg/L (18, 30). GA has other properties in common with naturally occurring antibiotic resistance-breaking AMPs, being cationic, <150 amino acid residues long, and exerting antimicrobial effects over an extremely short time course to a level similar to that of the AMP LL-37 (29, 31). GA can also form secondary structures and oligomerize (29, 32). In MS, the mechanisms of GA action are complex, multifactorial, and not fully understood, but it is known that GA acts as an immunomodulator, shifting T-helper cell populations, increasing secretion of anti-inflammatory cytokines, and killing leukocytes via membrane damage, among other properties (29, 33, 34).

Investigating existing drugs which are not conventional antibiotics for their potential use as antimicrobials, known as “repurposing,” is a growing area of interest and research (35, 36). As well as addressing the paucity of new antibiotics being developed, repurposing of existing drugs has several other advantages: approved drugs have historical safety data, and repurposing may minimize time and costs associated with deployment to the clinic due to the availability of previous trials and research (36).

As with other members of the aminoglycoside family, tobramycin targets the bacterial protein synthesis machinery and thus requires uptake into the bacterial cytoplasm for activity (37). Given its widespread use in CF, preserving, restoring, and/or enhancing the efficacy of tobramycin is of particular interest and importance. As it has also been previously subject to “breaking” by antimicrobial peptides, we examined tobramycin in combination with GA to assess the peptide as a potential antibiotic resistance breaker in cystic fibrosis strains of P. aeruginosa. We quantified the impact of GA on bacterial survival over a range of tobramycin concentrations and assessed its direct effects on outer and cytoplasmic membranes to elucidate mechanisms of action.

## RESULTS

### Combining glatiramer acetate and tobramycin alters growth and viability of Pseudomonas aeruginosa.

**FIG 1:**
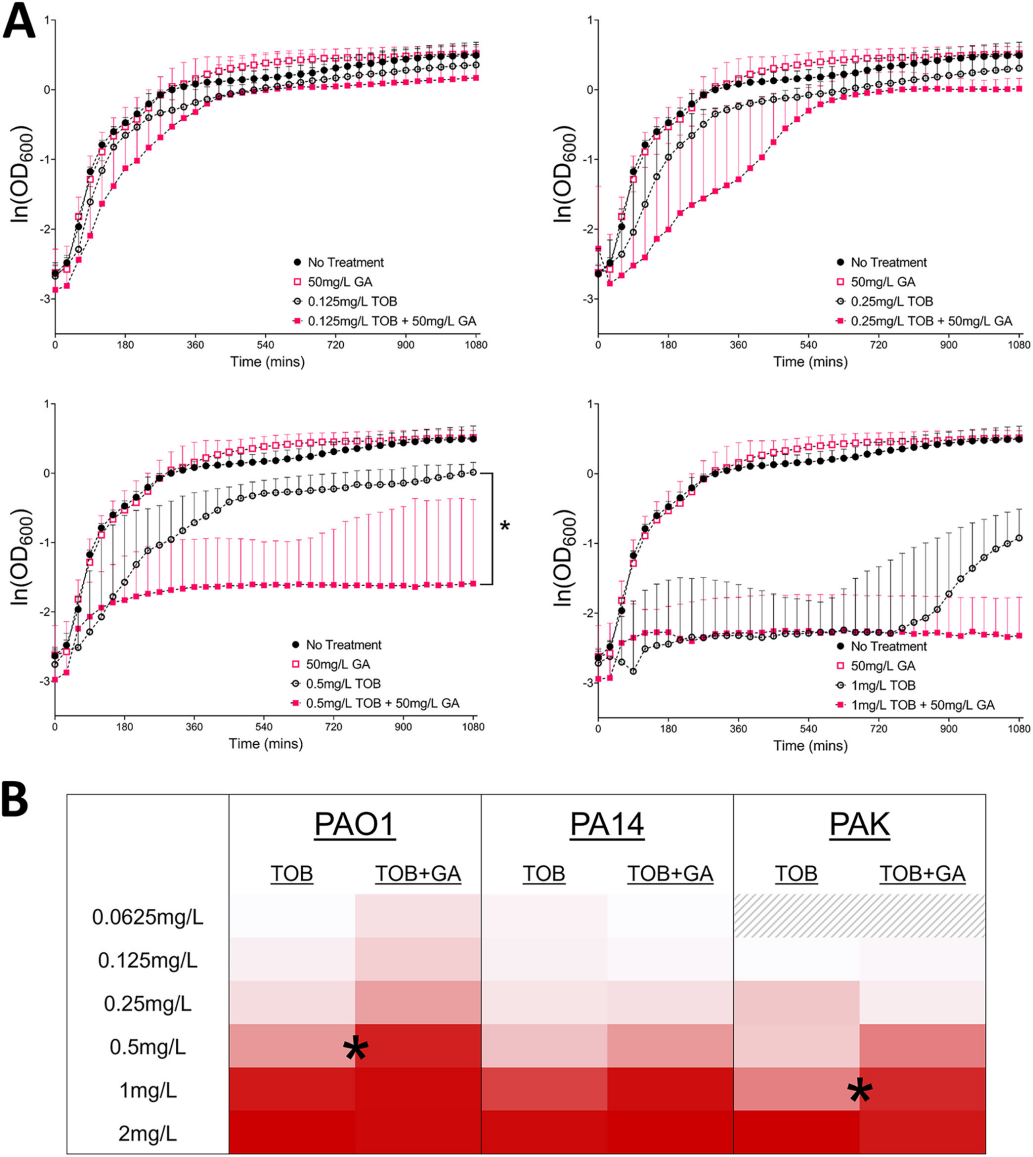
(A) Semilog plots of overnight growth of P. aeruginosa PAO1 (natural logs of the OD_600_, with medians and 95% confidence intervals) in increasing concentrations of TOB, with and without GA. Combination effect of GA plus TOB was noted at 0.5 mg/L TOB, where the final OD_600_ of the growth curves was significantly lower for GA with TOB than for TOB alone (*P* = 0.0159). (B) Heat map of AUC data for PAO1, PA14, and PAK. OD_600_ values for overnight growth curves at all TOB concentrations tested are shown (medians of at least three biological replicates). Darkness of red indicates a lower AUC and less bacterial growth. For PAO1 and PAK, GA with TOB resulted in significantly lower AUCs than did TOB alone at 0.5 and 1 mg/L, respectively (*P* = 0.0317 for each).

We anticipated that if GA possessed antibiotic potentiation properties, this would likely be demonstrable only with certain concentrations of TOB; in the presence of an effective concentration (above the strain-specific MIC) of antibiotic, any impact of GA could not be visualized in these experiments, whereas presumably too low a concentration of antibiotic could limit the impact of a compound capable of potentiation. We first tested this with a relatively high-throughput approach using growth curves generated from serial measurements (based on the optical density at 600 nm [OD_600_]) during overnight culture of PAO1, PA14, and PAK exposed to a range of tobramycin concentrations (0.06 to 4 mg/L in log_2_ increments) with and without 50 mg/L GA. No strain was inhibited by GA alone, and the concentration of TOB alone which impacted growth differed across strains, PAO1 being the most susceptible and PAK the least (Fig. 1; see also Fig. S1 in the supplemental material). Confirming our hypothesis, for each strain we could identify TOB concentrations at which GA potentiated its activity.

For strain PAO1, this concentration was 0.5 mg/L TOB (Fig. 1A). The median OD_600_ at 18 h for PAO1 cultures treated with GA and TOB (0.5 mg/L) was significantly lower than that of cultures treated with this concentration of TOB alone: 0.21 (95% confidence interval [CI], 0.17 to 0.78) versus 1.11 (95% CI, 0.74 to 1.35; *P* < 0.05). The area under the curve (AUC) analyses confirmed this significant effect (*P* < 0.05) (Fig. 1B). For strain PAK, AUC analysis demonstrated potentiation of TOB by GA at 1 mg/L (*P* < 0.05) (Fig. 1B; see also Fig. S1 in the supplemental material). While similar analyses for PA14 did not reach statistical significance, a visual trend suggestive of potentiation was observed, also at 1 mg/L TOB (see Fig. S1).

**FIG 2:**
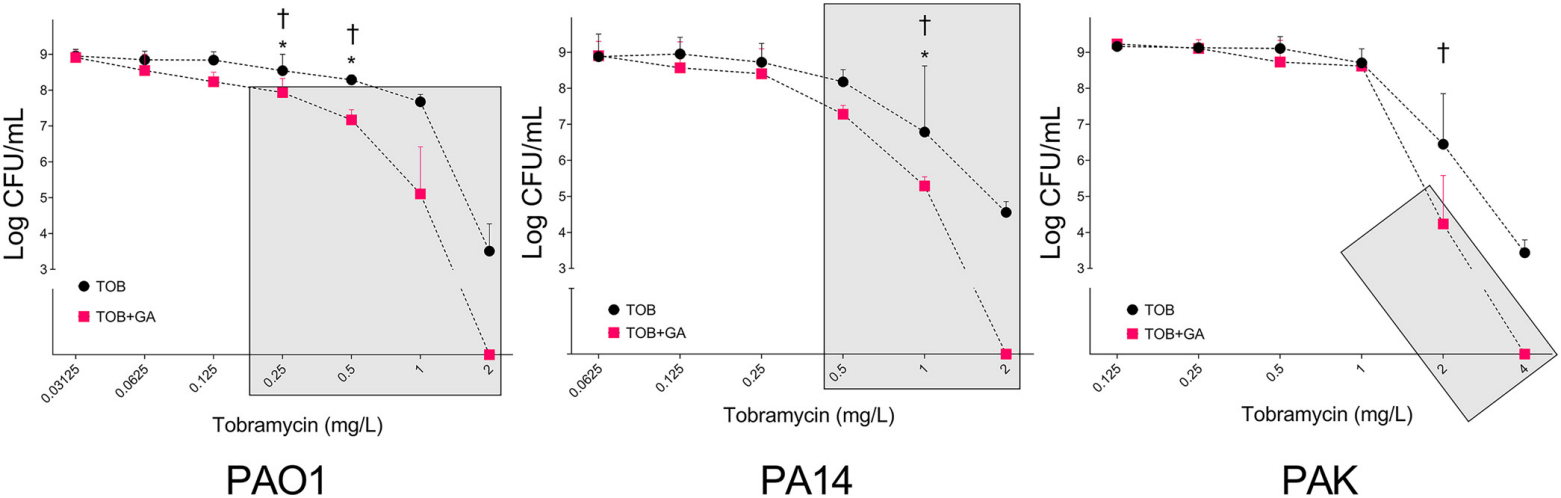
CFU of strains PAO1, PA14, and PAK after overnight exposure to TOB or GA with TOB at increasing TOB concentrations (medians with 95% confidence intervals). *, concentrations for which GA plus TOB resulted in significantly fewer viable bacteria than did TOB alone (for PAO1 at 0.25 mg/L [*P* = 0.026] and at 0.5 mg/L [*P* = 0.0286]; for PA14 at 1 mg/L [*P* = 0.0286]). †, the TOB concentrations where GA plus TOB displayed synergy (E_OBs_ > E_Exp_). Shaded areas indicate results (in CFU per milliliter) which were significantly lower than for untreated P. aeruginosa (data not shown on graphs; i.e., a stable CFU value as seen with the lowest concentrations of TOB) for a given strain (*P* < 0.05). For each strain, the highest GA plus TOB concentration tested eliminated viable bacteria, whereas the same TOB-only treatment did not. At least three biological replicates were performed.

Next, we sought to confirm and extend the observations from growth curve assays by colony counting. Viable colony counts were performed on overnight cultures after treatment with GA, TOB, or GA and TOB with at least three biological replicates at each antibiotic concentration. GA alone did not affect CFU counts of any strain and, consistent with the findings above, CFU were reduced more by GA and TOB cotreatment than by TOB alone (Fig. 2). Of note, a significant impact was observed over a greater range of TOB concentrations than were observed with growth curve screening.

For PAO1, combining GA with either 0.25 or 0.5 mg/L TOB resulted in significantly fewer colonies than results with the same concentration of TOB only: 8.54 × 10^7^ (95% CI, 1.38 × 10^7^ to 2.10 × 10^8^) versus 3.48 × 10^8^ (95% CI, 1.41 × 10^8^ to 1.00 × 10^9^) and 1.49 × 10^7^ (95% CI, 1.66 × 10^6^ to 2.85 × 10^7^) versus 1.94 × 10^8^ (95% CI, 1.33 × 10^8^ to 2.65 × 10^8^), respectively (*P* < 0.05 for both comparisons). For both of these GA-TOB combinations, numerically fewer viable bacteria were recovered than with the 2-log-higher TOB concentration administered alone (Fig. 2). Furthermore, these TOB concentrations used alone did not significantly reduce the CFU count per milliliter compared with untreated cultures, whereas both GA and TOB concentrations had highly significant impacts (*P* < 0.01) (Fig. 2). For PA14, a significant impact of the addition of GA was seen at 1 mg/L TOB, with >1 log_10_ fewer CFU per milliliter: 1.94 × 10^5^ (95% CI, 2,050 to 3.50 × 10^5^) versus 6.10 × 10^6^ (95% CI, 4.80 × 10^6^ to 4.19 × 10^8^; *P* < 0.05) (Fig. 2). The impact on PAK was visually similar but did not reach statistical significance (Fig. 2).

We further analyzed these CFU data to assess whether any impact of GA on TOB was additive or synergistic, with the latter defined as the combined effect of the two agents being greater than would be predicted by the individual effects for each (E_Obs_ > E_Exp_). For PAO1, the combination of GA with either 0.25 mg/L or 0.5 mg/L TOB was synergistic; for PA14, synergy was observed for GA with 1 mg/L TOB; for PAK, GA with 2 mg/L TOB was synergistic (Fig. 2; see also Table S2 in the supplemental material).

### Glatiramer acetate reduces the MICs of tobramycin against reference strains of P. aeruginosa.

**FIG 3:**
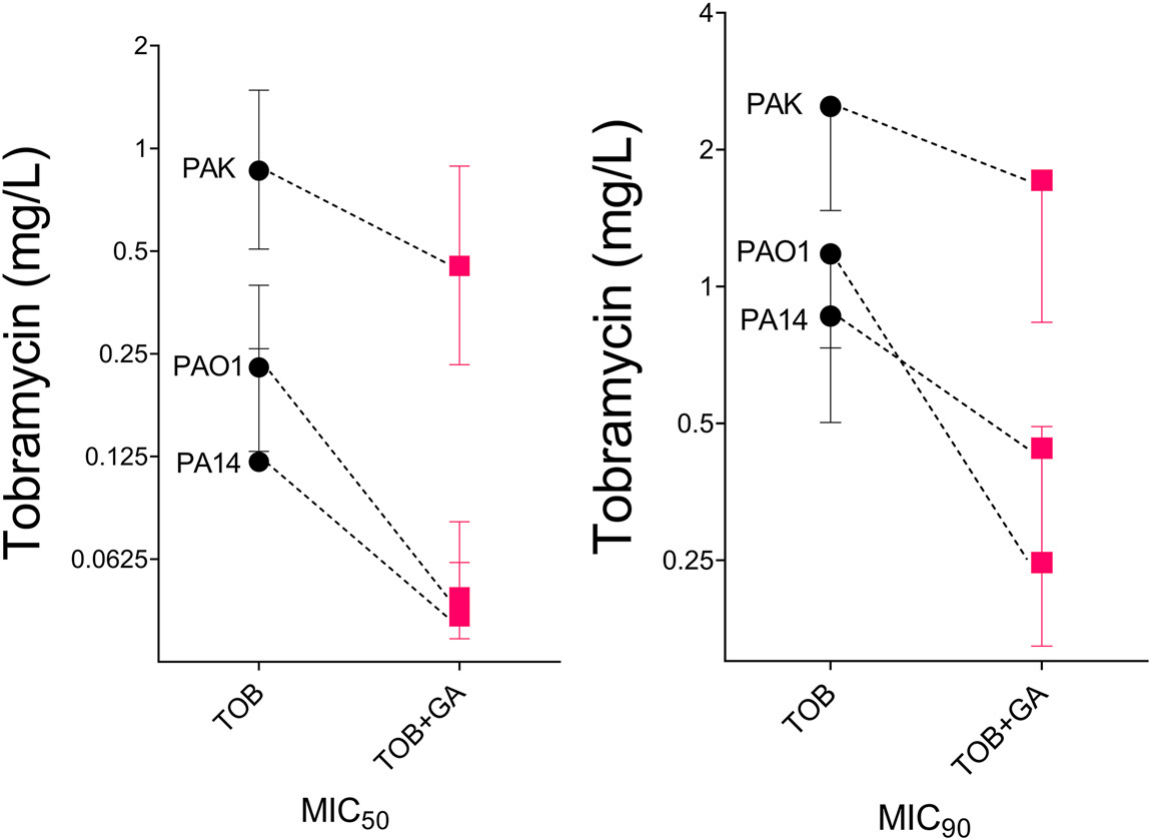
MIC_50_ and MIC_90_ values of TOB for strains PAO1, PA14, and PAK in the absence and presence of GA. Interpolated MIC values and their 95% confidence intervals are shown (from nonlinear fit of inhibition curves, in CFU per milliliter results derived using results from at least 3 biological replicates).

For each of the three reference strains, colony count results were used to calculate the inhibition of P. aeruginosa (as a percentage of results in untreated cultures) caused by TOB and GA plus TOB, at each TOB concentration tested, and inhibition curves were generated (Fig. 3). From the resulting fitted curves, the MIC_50_ and MIC_90_ levels of tobramycin were interpolated when GA was present and absent (see Fig. S2 in the supplemental material).

Fold reductions in the MIC_50_ of 4.7, 2.8, and 1.9 and in the MIC_90_ of 4.7, 1.9, and 1.5 for PAO1, PA14, and PAK, respectively, were observed (Fig. 3; see also Table S1 in the supplemental material).

### Glatiramer acetate reduces the MICs of tobramycin for clinical cystic fibrosis strains of P. aeruginosa.

Eleven clinical isolates of P. aeruginosa from people with cystic fibrosis were selected (Table 1). As with the reference strains, clinical isolates were screened using growth curves, colony counts were performed, and MIC values were interpolated from inhibition curves for each isolate (at least three biological replicates were performed). The sensitivity results generated for tobramycin via this method were consistent with the disc diffusion results reported by the clinical lab, using the EUCAST MIC breakpoint of 4 mg/L for tobramycin.

**TABLE 1:** Clinical P. aeruginosa isolates used in this study from people with cystic fibrosis at Royal Brompton Hospital, London

Strain	Sample type	Mucoidy	Multidrug resistance	Tobramycin	Ceftazidime	Ciprofloxacin	Colistin	Meropenem
RBH550	Spontaneous sputum			Sensitive	Sensitive	Sensitive	Sensitive	Sensitive
RBH461	Cough swab		MDR	Resistant	Resistant	Resistant	Sensitive	Resistant
RBH490	Spontaneous sputum		MDR	Resistant	Resistant	Resistant	Sensitive	Resistant
RBH519	Spontaneous sputum	Mucoid		Resistant	Sensitive	Resistant	Sensitive	Sensitive
RBH072	Spontaneous sputum			Resistant	Sensitive	Resistant	Sensitive	Sensitive
RBH294	Spontaneous sputum	Mucoid		Sensitive	Sensitive	Sensitive	Sensitive	Sensitive
RBH899	Spontaneous sputum			Sensitive	Sensitive	Sensitive	Sensitive	Sensitive
RBH750	Spontaneous sputum	Mucoid		Sensitive	Sensitive	Sensitive	Sensitive	Sensitive
RBH065	Spontaneous sputum			Resistant	Sensitive	Resistant	Sensitive	Sensitive
RBH422	Spontaneous sputum			Sensitive	Sensitive	Resistant	Resistant	Sensitive
RBH982	Spontaneous sputum			Sensitive	Sensitive	Sensitive	Sensitive	Sensitive

**FIG 4:**
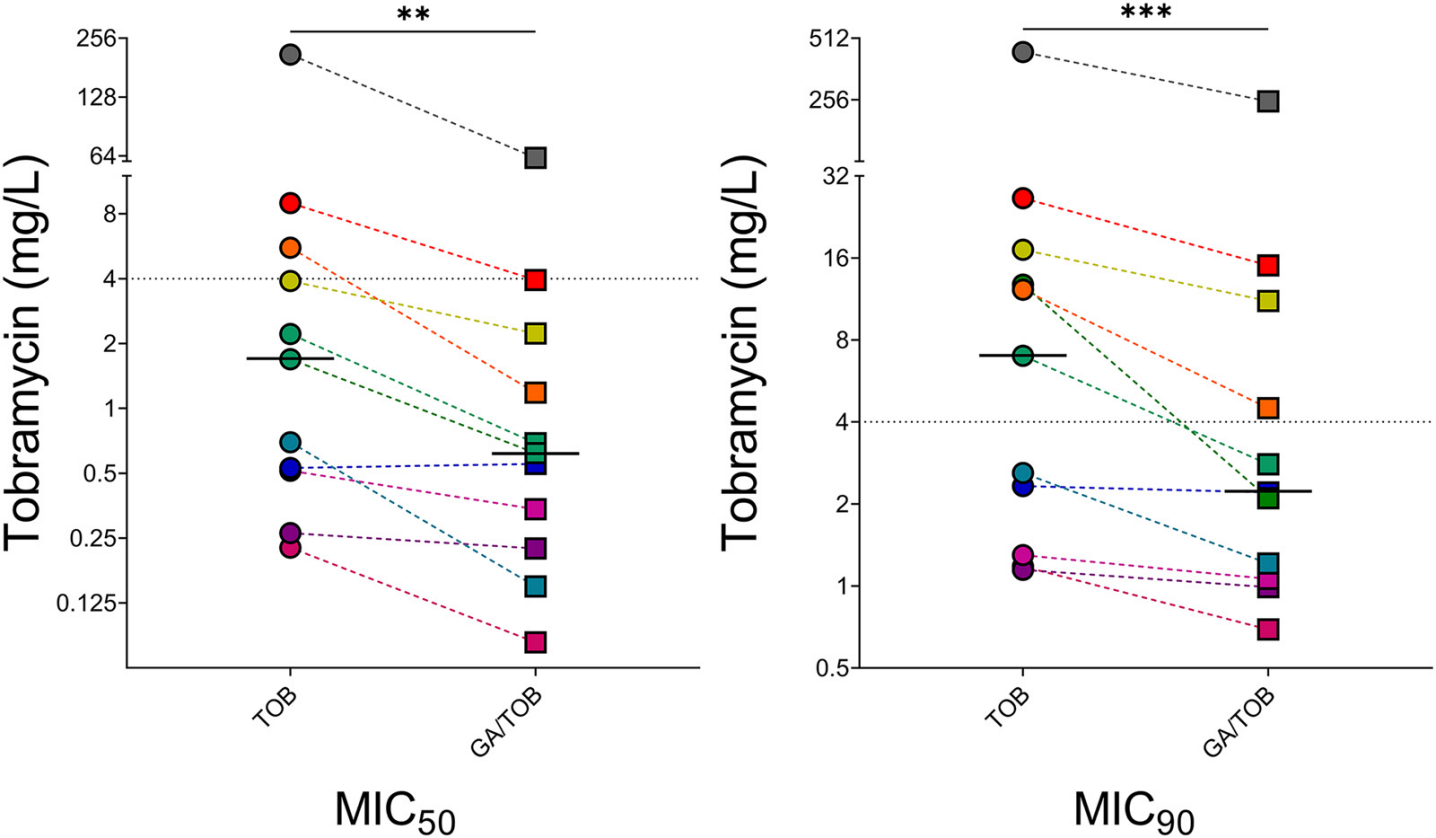
MIC_50_ and MIC_90_ results for TOB, in the absence and presence of GA, for 11 clinical P. aeruginosa strains from cystic fibrosis patients. Values were interpolated from nonlinear fit of inhibition curves (CFU per milliliter) from at least 3 biological replicates for each strain. Across the 11 strains tested, both MIC_50_ and MIC_90_ values of TOB were significantly reduced by coadministration of GA (*P* = 0.002 and *P* = 0.001, respectively).

Across the whole panel of 11 CF clinical P. aeruginosa isolates tested here, GA significantly reduced both the MIC_50_ (median; from 1.69 mg/L [95% CI, 0.26 to 8.97] to 0.62 mg/L [95% CI, 0.15 to 3.94]; 2.7-fold reduction; *P* = 0.002) and MIC_90_ (median; from 7.00 mg/L [95% CI, 1.18 to 26.50] to 2.20 mg/L [95% CI, 0.99 to 15.03]; 1.7-fold reduction; *P* = 0.001) of tobramycin (Fig. 4; see also Table S1). Synergy analyses based on these results indicated that for 6 of the 11 clinical strains tested, there was at least one TOB concentration at which the antibiotic acted synergistically with GA, with the remainder having at least one TOB concentration for which GA was additive (see Table S2). Importantly, no antagonism was seen for any isolate at any concentration of TOB. Both mucoid and nonmucoid strains were included for testing, and no impact of mucoidy was noted, albeit a relatively small number of strains were tested.

The 6 TOB-sensitive P. aeruginosa strains had a median MIC_50_ for TOB alone of 0.52 mg/L (95% CI, 0.23 to 2.21), which in the presence of GA was 0.28 (95% CI, 0.08 to 0.69) (not significant). Similarly, for these strains the MIC_90_ was 1.81 mg/L (95% CI, 1.15 to 7.00) for TOB alone and 1.14 mg/L (95% CI, 0.69 to 2.80) for GA plus TOB (*P* < 0.05). GA was not effective in reducing tobramycin MICs for every sensitive strain tested; one strain, RBH294, showed similar results for TOB effectiveness whether GA was present or not.

The 5 tobramycin-resistant P. aeruginosa clinical isolates also demonstrated reductions in effective concentrations of tobramycin when GA was coadministered. The tobramycin MIC_50_ of this group was reduced by GA from 5.57 mg/L (95% CI, 1.69 to 211.19) to 2.23 mg/L (95% CI, 0.62 to 62.50), and the MIC_90_ fell from 17.2 mg/L (95% CI, 12.23 to 438.32) to 11.11 mg/L (95% CI, 2.10 to 251.49) (neither of which reached statistical significance for this subgroup). GA reduced the tobramycin MIC_50_ by 2.72-fold (95% CI, 1.75 to 4.70) and the MIC_90_ 1.76-fold (95% CI, 1.54 to 6.08). For 4 of the 5 tobramycin-resistant strains tested, cotreatment with GA resulted in the MIC_50_ being decreased to <4 mg/L, the EUCAST breakpoint concentration of tobramycin, indicating these strains would be considered tobramycin-sensitive in the presence of GA.

### Glatiramer acetate disrupts the outer bacterial membrane of P. aeruginosa.

**FIG 5:**
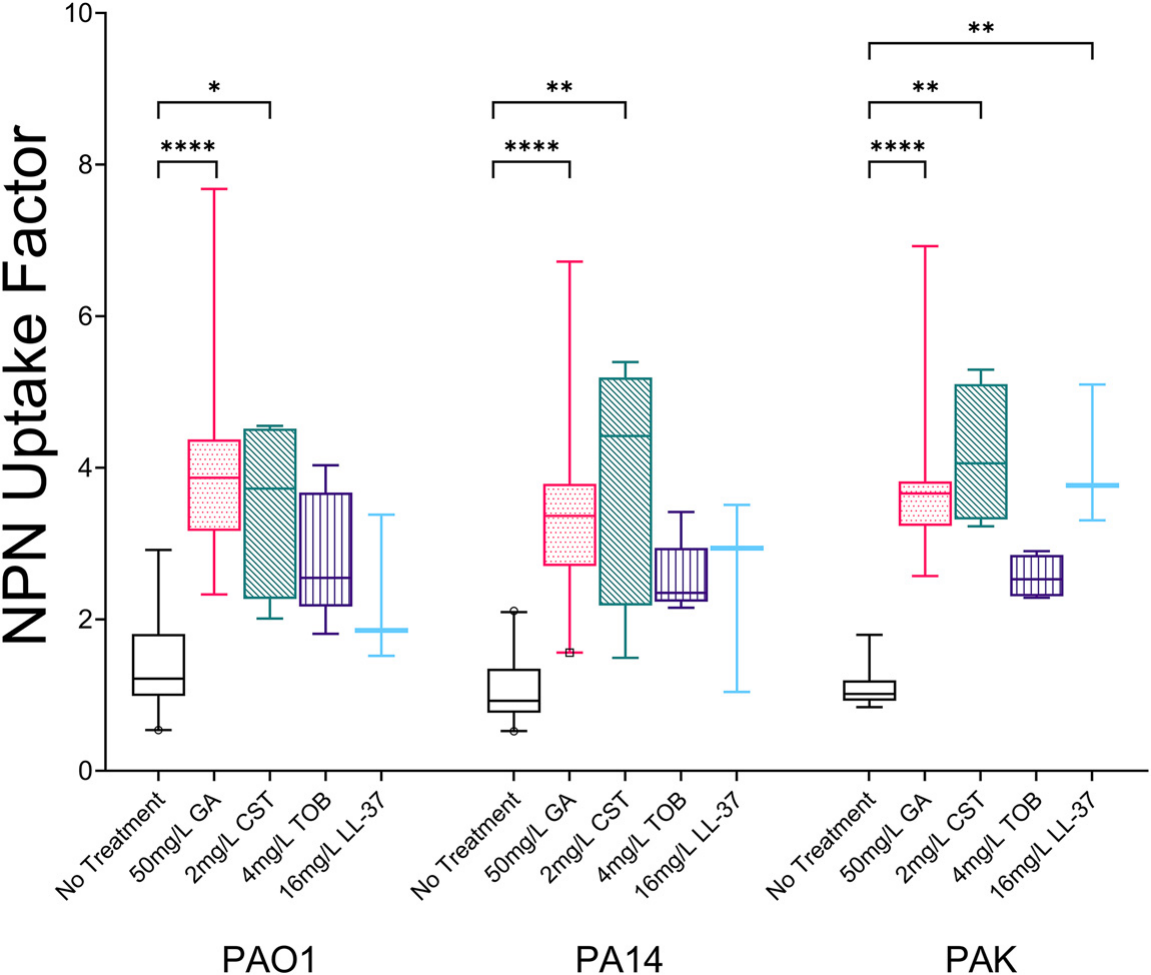
Comparison of uptake of NPN over 15 min by the outer membranes of strains PAO1, PA14, and PAK resulting from exposure to 50 mg/L GA, 2 mg/L CST, 4 mg/L TOB, or 16 mg/L LL-37 (box and whisker plot, line at median with 5th and 95th percentiles). At least three biological replicates of each treatment were performed for each strain. GA caused a significant increase in outer membrane disruption for all three reference strains (each strain, *P* < 0.0001). CST significantly increased NPN uptake in each strain (PAO1, *P* = 0.0101; PA14, *P* = 0.001; PAK, *P* = 0.0012), while LL-37 increased NPN uptake in PAK (*P* = 0.0037).

We next wished to explore the mechanism by which GA potentiated the activity of TOB. The fluorescent probe 1-*N*-phenylnaphthyamine (NPN) was used to measure disruption of the outer membrane of P. aeruginosa strains. Increased fluorescence indicates NPN is binding to the inner hydrophobic elements of the outer membrane, which are only accessible in disrupted membranes. Treatment with GA significantly increased median uptake of NPN by PAO1, PA14, and PAK over 15 min. GA resulted in a 3.43-fold increase (95% CI, 3.28 to 3.49) in NPN uptake by reference strains of P. aeruginosa, compared with that in untreated cultures (*P* < 0.0001) (Fig. 5). Comparison with other antimicrobials showed that uptake of NPN from GA treatment was comparable with treatment with colistin (CST; 2 mg/L; 3.99-fold increase [95% CI, 3.71 to 4.79]) and the membrane-disrupting peptide LL-37 (16 mg/L; 3.70-fold increase [95% CI, 2.74 to 3.80]), while treatment with the aminoglycoside antibiotic tobramycin resulted in a 2.14-fold increase (95% CI, 1.97 to 2.55) in the NPN uptake factor compared to that for untreated P. aeruginosa cells (Fig. 5).

**FIG 6:**
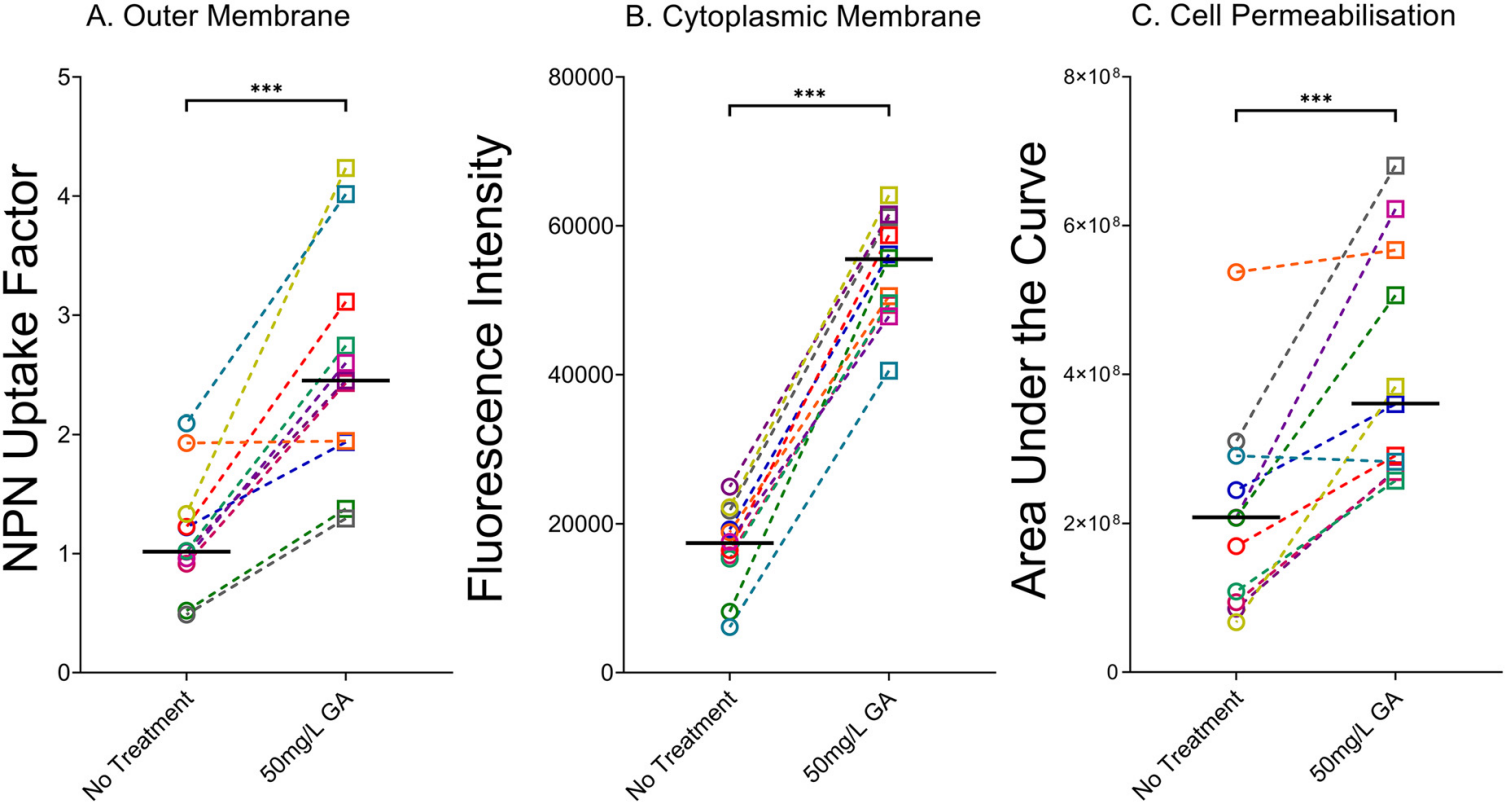
Effect of 50 mg/L GA on the outer membranes (A), cytoplasmic membranes (B), and permeability (C) of 11 cystic fibrosis P. aeruginosa strains. Each point represents a strain and is the median of at least three independent experiments. Line is at median of all clinical strains tested. GA caused significant disruption of bacterial outer membranes, significant depolarization of the cytoplasmic membrane, and significant permeabilization of the cell wall, compared to untreated bacteria (*P* = 0.001, *P* = 0.001, and *P* = 0.002, respectively). Responses for individual strains were variable, ranging from none to a high magnitude of change. For this reason, we display data for individual isolates.

Glatiramer acetate caused a significant increase in the uptake of NPN in the outer membranes for the group of 11 clinical P. aeruginosa strains from CF (median untreated, factor of 1.02 [95% CI, 0.52 to 1.93], to GA-treated, factor of 2.45 [95% CI, 1.38 to 4.02]; *P* = 0.001) (Fig. 6A).

### Glatiramer acetate depolarizes the cytoplasmic membrane of P. aeruginosa.

**FIG 7:**
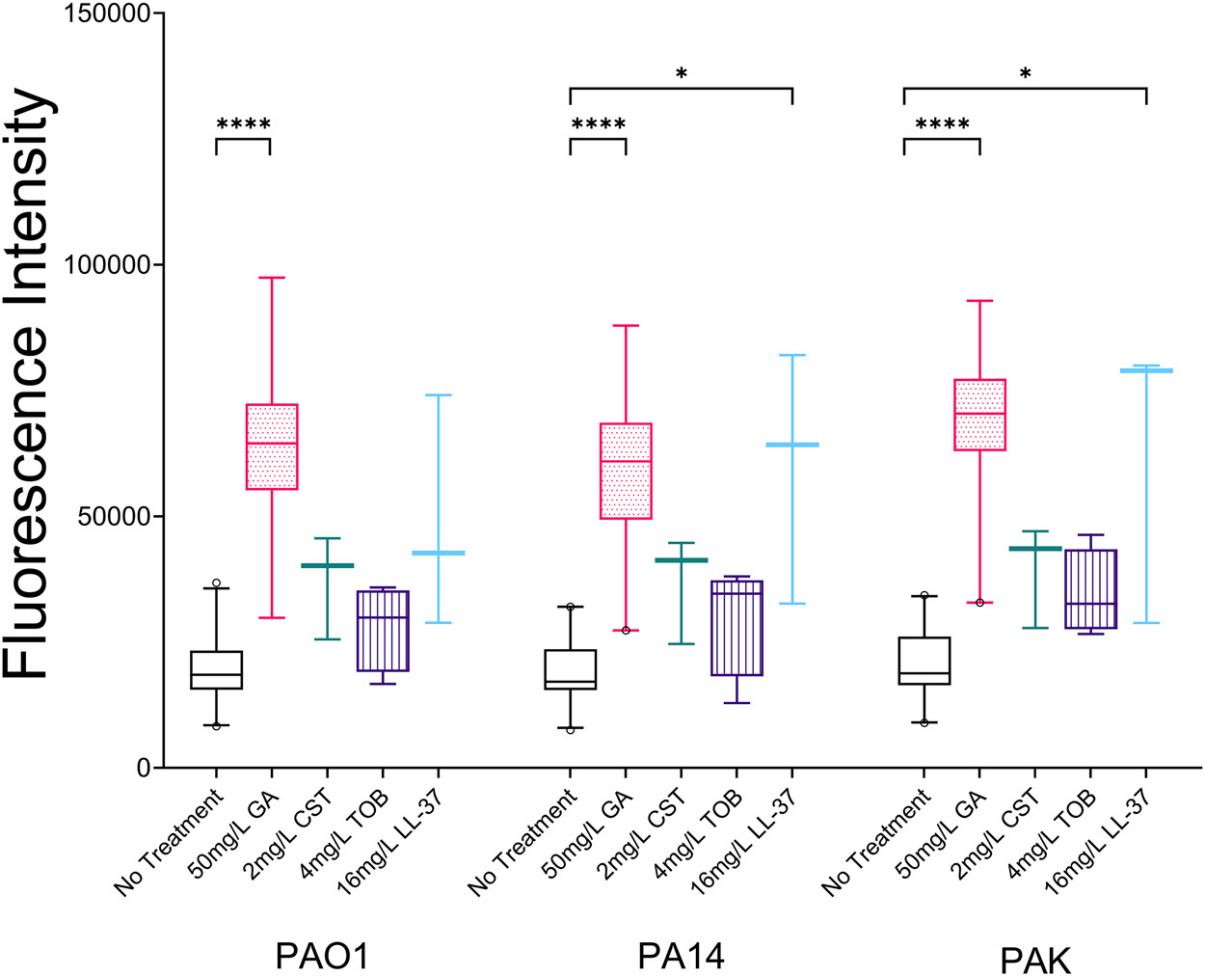
Comparison of release of DiSC_3_(5) over 15 min by the cytoplasmic membranes of strains PAO1, PA14, and PAK resulting from exposure to 50 mg/L GA, 2 mg/L CST, 4 mg/L TOB, or 16 mg/L LL-37 (box and whisker plot, line at median with 5^th^ and 95 the percentiles). At least three biological replicates of each treatment were performed for each strain. GA caused a significant depolarization of cytoplasmic membranes for all three reference strains (each strain, *P* < 0.0001). Depolarization was also seen for LL-37 treatment of PA14 (*P* = 0.0122) and PAK (*P* = 0.0152).

The fluorescent dye 3,3′-dipropylthiadicarbocyanine Iodide [DiSC_3_(5)] was used to measure depolarization of the cytoplasmic membrane of P. aeruginosa. On application, the dye integrates into the intact cytoplasmic membrane; any subsequent intervention causing membrane depolarization will lead to dye release and increased fluorescent signal. Treatment with GA resulted in rapid release of DiSC_3_(5) by PAO1, PA14, and PAK, with a higher fluorescent signal produced than by TOB or CST. Examining the median fluorescence over the first 15 min of treatment showed that GA resulted in each of the three P. aeruginosa reference strains releasing significantly more DiSC_3_(5) than the untreated cultures (*P* < 0.0001), while of the other agents tested, only LL-37 (16 mg/L) caused significant cytoplasmic membrane depolarization of PA14 and PAK (Fig. 7).

A similar effect was seen for the 11 clinical CF P. aeruginosa strains. Median DiSC_3_(5) release over 15 min was 3.19 times higher (95% CI, 2.68 to 6.32) with 50 mg/L GA than with no treatment (*P* = 0.001). Treatment with GA resulted in a minimum increase in dye release of 2.65-fold (95% CI, 2.13 to 2.96), while the highest level seen for any clinical strain was a 6.81-fold increase over untreated (95% CI, 2.75 to 7.06) (Fig. 6B).

### Glatiramer acetate permeabilizes P. aeruginosa cells.

**FIG 8:**
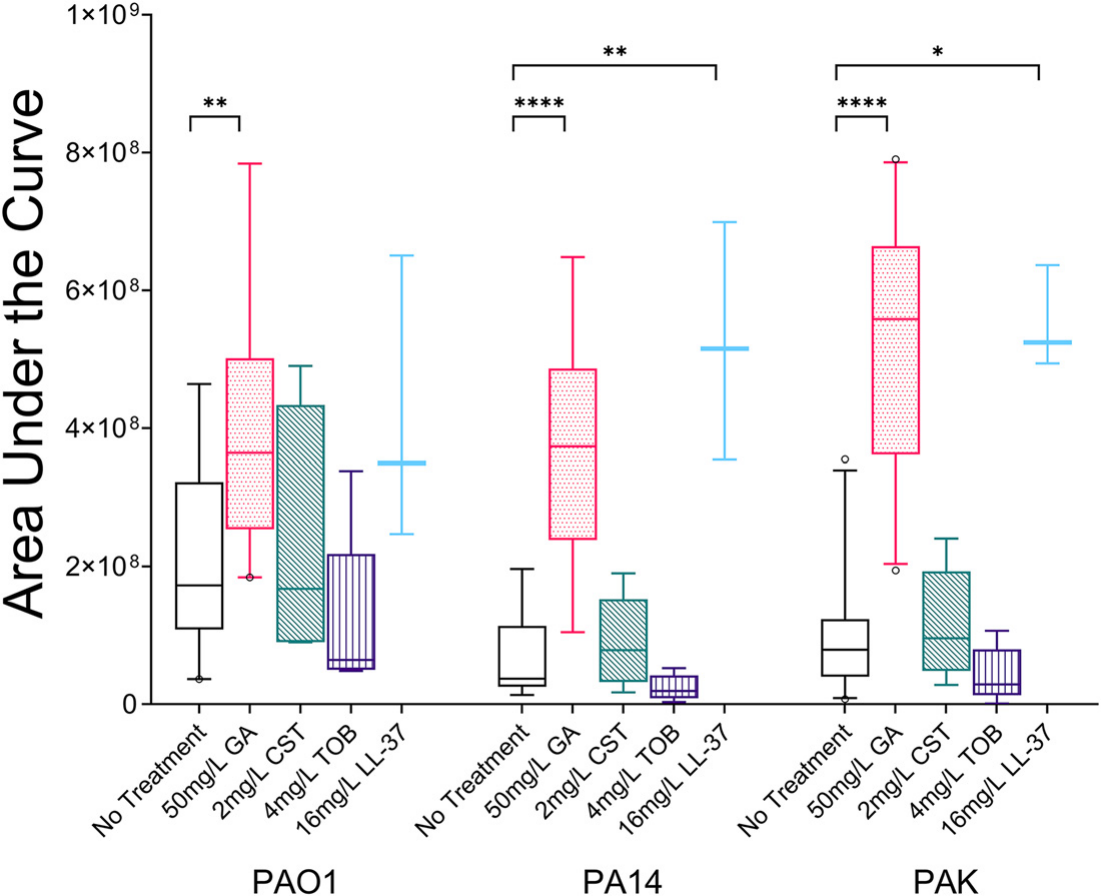
Comparison of AUC data for propidium iodide fluorescence over 1 h with strains PAO1, PA14, and PAK resulting from exposure to 50 mg/L GA, 2 mg/L CST, 4 mg/L TOB, or 16 mg/L LL-37 (box and whisker plot, line at median with 5th and 95th percentiles). At least three biological replicates of each treatment were performed for each strain. GA caused a significant increase in bacterial cell permeabilization of all three reference strains (for PAO1, *P* = 0.0087; for PA14 and PAK, *P* < 0.0001). Permeabilization was also seen for LL-37 treatment of PA14 (*P* = 0.0073) and PAK (*P* = 0.0248).

Having noted the ability of GA to perturb both the outer and cytoplasmic membranes of P. aeruginosa, the DNA-binding membrane-impermeable dye propidium iodide (PI) was used to test whether the bacteria were permeabilized by GA. Each P. aeruginosa reference strain was significantly permeabilized by 50 mg/L GA compared to untreated cultures, as assessed by AUCs of 1 h PI fluorescence (*P* < 0.001) (Fig. 8). None of the other agents resulted in permeabilization, with the exception of LL-37 (PA14 [*P* < 0.001] and PAK [*P* < 0.05]) (Fig. 8). For the clinical strains, GA resulted in a 2.37-fold increase (95% CI, 1.05 to 3.18) in permeabilization (*P* = 0.002 versus untreated cells) (Fig. 6C).

## DISCUSSION

We hypothesized that the peptide drug glatiramer acetate would be an antibiotic resistance breaker in P. aeruginosa when combined with the aminoglycoside tobramycin. Here, we provide data to support this hypothesis and demonstrate the drug’s mechanism of action, using a single concentration of GA previously shown to be optimally effective against P. aeruginosa (18). Initial tests with a small number of reference strains revealed that GA potentiated the activity of tobramycin, as seen by bacterial growth, viability, and MICs for these strains. Expanding these tests to clinical P. aeruginosa isolates from people with cystic fibrosis confirmed these observations and resulted in significant reductions of TOB MICs; in the majority of TOB-resistant strains, the presence of GA rendered them TOB-sensitive. These effects likely relate to GA’s interaction with and disruption of the cell envelope of P. aeruginosa strains, causing damage to the outer membrane, depolarization of the cytoplasmic membrane, and permeabilization of the bacteria.

We employed a wide variety of techniques with both reference and clinical strains to fully understand the impact of GA. Using colony counts to calculate precise MICs, we observed that GA was able to lower the MIC_50_ and MIC_90_. GA acted synergistically with tobramycin in the treatment of the 3 reference strains; the reduction in viable bacteria resulting from cotreatment was greater than would be expected, based on the individual treatments. We analyzed synergy using Bliss independence which, while less commonly used, allows for synergy testing in agents with differing mechanisms of action (such as GA and tobramycin) and also allows for the comparison of observed and expected results of agent combinations without the use of checkerboarding (38, 39).

The ability to enhance or recover the activity of existing antibiotics is an extremely attractive property in a drug. An agent which is also already in clinical use and has moderate antibacterial activity is potentially desirable as a potential antibiotic adjuvant. It is crucial that studies such as this include clinical isolates, which are known to differ substantially from reference strains at both genetic and phenotypic levels. There is no gold standard for clinically significant reductions in colony numbers due to the well-documented difficulties in accurately enumerating bacterial loads in infections, particularly in the heterogenous respiratory system and further confounded in the CF lung. Our findings are of 1- and 2-log reductions in CFU counts per milliliter after combined treatments and significant reductions at strain-specific tobramycin concentrations. In the pivotal phase 3 clinical trials of tobramycin (current standard of care in CF P. aeruginosa infection), reductions in P. aeruginosa CFU counts per milliliter of 0.8 log were reported (40).

Encouragingly, GA plus TOB synergy was also seen in the majority of strains in clinical P. aeruginosa from people with cystic fibrosis tested. This was the case regardless of whether the individual strain had been designated sensitive or resistant to tobramycin by standard clinical laboratory disc diffusion testing. The isolates with both the lowest and highest MICs of tobramycin were more susceptible to the aminoglycoside when GA was also present. This was not the case for every clinical isolate tested, and we found examples of clinical strains which were unaffected in their sensitivity to tobramycin. Interestingly, this was not related to the resistance profiles of these strains; both tobramycin-sensitive and -resistant P. aeruginosa isolates were among those with MICs minimally or not altered by cotreatment. While the methodology used allowed for the calculation of both MIC_50_ and MIC_90_ to a fine degree for each strain and allowed comparisons across the clinical strain panel, it limited the scope to a small sample number. Future work will focus on what these strains have in common, which may allow them to resist the potentiation of tobramycin by GA. Our data would appear to indicate that the two agents have distinct mechanisms of action given their ability to synergize, the lack of correlation between synergy and susceptibility to tobramycin, and contrasting results from the two drugs in membrane perturbation assays.

Intrinsic antibiotic resistance in P. aeruginosa is closely related to the bacterium’s ability to exclude compounds from its cytoplasm, either via physical barrier (membrane impermeability) or toxin removal (efflux pumps). P. aeruginosa’s membrane is less permeable than that of other Gram-negative pathogens, e.g., Escherichia coli (41–43). Indeed, in P. aeruginosa isolates from cystic fibrosis patients, “impermeability resistance” has been found to be the most common form of resistance to tobramycin (44, 45). P. aeruginosa also encodes in its genome efflux pumps, such as MexXY, which can remove antibiotics, including tobramycin, from the cell interior (45–47). The concentration of GA used in this study (and higher concentrations) did not affect the overnight growth of P. aeruginosa, suggesting that there is no selection for resistance. There is also evidence that antimicrobial peptides, such as GA, are less likely to result in resistance than other antibiotic classes; they do not increase recombination rates (48, 49), and colistin resistance was slower to emerge than resistance to other antibiotics (50).

In common with many other antimicrobial peptides, we found that GA disrupts the outer bacterial membrane, depolarizes the cytoplasmic membrane and, ultimately, permeabilizes the P. aeruginosa cell, and we hypothesized that this property results in potentiation of TOB. The results reported here show that GA is as effective or more effective at causing membrane perturbations in P. aeruginosa reference strains than are the peptides colistin and LL-37. We chose concentrations of CST and LL-37 which are known to be active against P. aeruginosa as comparators, and we think it is of great interest that GA, at the concentrations tested, was more active across membrane disruption assays (despite not having a conventional MIC) than MICs of CST or LL-37, even though there was a disparity in the specific concentrations of each tested. Among the agents tested, GA was the only treatment capable of causing significant changes to both membranes, which would allow entry to the cytoplasm, in all reference strains. Aminoglycosides have intracellular targets (ribosomal binding sites) where they can prevent protein production and cell replication (51). The activity of GA in disrupting the bacterial membrane may allow tobramycin access to these targets within the bacteria, by breaching the normally impermeable cell wall and/or overwhelming the activity of efflux pumps, preventing the cell from removing the antibiotic as quickly as it accumulates. For clinical P. aeruginosa isolates, significant increases in membrane perturbations resulting from GA were also found across the group of strains for each test performed (Fig. 6). Increases were not seen for each individual strain tested and, indeed, strains had differential responses to GA across their membranes, which may indicate more than one distinct mechanism of GA activity, linked to the known heterogeneity of GA and ability to oligomerize and form secondary structures (29, 32, 52).

P. aeruginosa impermeability is aided by LPS on the bacterial cell surface. P. aeruginosa can use divalent cations (e.g., Mg^2+^ and Ca^2+^) from the environment to bind together and stabilize the LPS chains, forming an additional barrier to the bacterial membrane (53). In the absence of environmental cations or in response to certain stimuli, P. aeruginosa can also modify LPS chains, for example by addition of 4-amino-4-deoxy-L-arabinose (L-Ara4N), thereby changing the overall charge of the bacterial cell and making it more difficult for negatively charged antimicrobials (including tobramycin) to bind and enter the cell (54–56). Polycationic antimicrobials, such as GA, can displace the divalent cations which normally bind and stabilize LPS-LPS interactions and thus expose more of the bacterial membrane to external pressures and agents (57, 58). Indeed, in the case of colistin, this targeting of LPS continues beyond the outer membrane and into the cytoplasmic membrane (59). The significance of divalent cations and LPS and their interactions have been known for over 50 years; the ability to potentiate antibiotics through cation and/or LPS removal and weakening of the defense they provide was demonstrated with the chelator EDTA (60–64). This has made the desire to discover other, clinically safe “permeabilizers” which potentiate antibiotics a longstanding one. We have demonstrated here GA’s ability to both permeabilize and potentiate P. aeruginosa to antibiotic treatment. Work is ongoing to investigate the specific interactions between GA and LPS structures and elucidate if GA potentiation works via similar LPS interactions.

One of the leading drivers of antibiotic resistance and the reduction in effectiveness of existing antibiotics is the lack of development of new antibiotics. The crisis in the development of antibiotic resistance may also have been exacerbated by the COVID-19 pandemic, with secondary bacterial infections and poor antibiotic stewardship resulting in surges in antibiotic prescriptions (65, 66). In the face of the dearth of investment in new antibiotics coming to market, alternative strategies are desperately needed (13, 35). While there are important roles to be played by better testing, better antibiotic stewardship, and novel strategies (bacteriophage, etc.), these tactics do not directly address the effectiveness of currently available antibiotics. Resurrecting and/or improving the efficacy of clinically valuable antibiotics would be of great benefit, especially for an antibiotic as important as tobramycin. However, close attention will need to be paid to route of delivery of any novel treatment or adjuvant. Interestingly, GA has been successfully nebulized in an animal model, without adverse effects (30). People with cystic fibrosis already face a significant treatment burden (median of 10 current treatments), and nebulized antibiotics are considered among the most burdensome of those treatments (67). Therefore, the most direct route to the CF clinic for an antibiotic adjuvant may be conebulization with a partner antibiotic.

Antibiotic-resistant organisms being rendered “sensitive” due to the combination of therapies has obvious benefits to infected patients. Clinical benefits may include eradicating infections more quickly than with monotherapy (of both resistant and sensitive strains) and preventing chronic infections from becoming established. Ancillary benefits could accrue if potential antibiotic side effects were reduced with shorter treatment courses or lower doses. Tobramycin has been associated with both ototoxicity and nephrotoxicity in cystic fibrosis, in a population with an already very high treatment burden of antibiotics (67–69). Whether or not the combined use of an antibiotic and a cotherapy such as GA would also have beneficial impact on resistance patterns emerging with time requires further study. While much attention is, naturally, focused on the treatment of resistant bacteria, and we have reported MIC reductions and synergy with TOB-resistant P. aeruginosa here, our results also show that GA has similar synergistic and MIC-reducing capabilities with TOB-sensitive strains. This may also be clinically important due to the heterogenous nature of the lung and the wide variation seen with nebulized tobramycin delivered to different regions (70, 71). Reducing the concentration required to be effective against all P. aeruginosa types could be of benefit in the face of these concentration gradients within sputum, in eradicating new infections and perhaps aiding in the prevention of future development of resistant bacteria.

While further work is also required to ensure the effectiveness of GA in the complex environment of CF sputum and to formulate delivery to people with cystic fibrosis, the confluence of antimicrobial activity, tobramycin synergy, and current clinical use makes GA a promising candidate for repurposing as an antibiotic adjuvant.

## MATERIALS AND METHODS

### Pseudomonas aeruginosa isolates.

Reference P. aeruginosa strains PAO1, PA14, and PAK and 11 clinical P. aeruginosa isolates were used in this study. Clinical strains were selected from the CF Bacterial Repository at the National Heart and Lung Institute, Imperial College London; this collection consists of bacteria isolated from airway samples of people with cystic fibrosis at the Royal Brompton Hospital, London (Table 1). Bacteria were stored in Microbank vials (Pro-Lab Diagnostics) at −80°C. Clinical strains were selected with a range of antibiograms according to clinical lab results using EUCAST clinical breakpoints. Isolates were revived onto cetrimide agar (Merck) for confirmation of purity and grown overnight at 37°C followed by subculture onto LB agar (Merck). Single colonies were inoculated into Mueller-Hinton broth (Merck) and incubated overnight at 37°C with agitation at 200 rpm.

### Bacterial growth curves and colony counts.

Late-exponential-phase cultures of P. aeruginosa were harvested by centrifugation (3,500 × *g* for 15 min) and resuspended in fresh Mueller-Hinton broth. Tobramycin solutions and bacterial cultures were prepared in Mueller-Hinton broth at double the desired concentrations and then mixed with cultures 1:1. The starting bacterial OD_600_ was 0.05 (~5 × 10^6^ CFU/mL). Tobramycin concentrations were tested stepwise in log_2_ increments. Tobramycin-bacteria solutions were vortexed briefly and aliquoted in triplicate 200-μL volumes into a sterile 96-well plate (ThermoScientific). Glatiramer acetate (Biocon, India; >99% purity) was added to the remainder of the tobramycin-bacteria suspension to a final GA concentration of 50 mg/L, the concentration previously identified as optimal in nondividing cultures over a short exposure (18). The three reference strains were tested for GA MICs in a conventional broth microdilution, and no inhibition was observed up to 1,600 mg/L GA (data not shown). Samples were vortexed and aliquoted to 3 further wells of the 96-well plate, also in 200-μL volumes. Untreated bacteria, bacteria treated with 50 mg/L GA only, and uninoculated Mueller-Hinton wells were also included. Plates were sealed with optically clear, breathable seals (4titude) and incubated in a FLUOstar Omega plate reader (BMG Labtech). The OD_600_ was measured every 30 min, overnight at 37°C with shaking at 200 rpm.

After overnight growth, triplicate wells were pooled and centrifuged, supernatant was removed, and pellets were resuspended in sterile phosphate-buffered saline (PBS; Merck). Serial 1:10 dilutions were performed in sterile PBS, and diluted cultures were spotted onto LB agar plates using the Miles-Misra method; 20-μL volumes with ≥10 technical repeats per condition were plated (72). Plates were allowed to dry at room temperature and placed in a 37°C incubator overnight. Spots with between 2 and 20 individual colonies were quantified. At least three biological replicates were performed.

### Outer bacterial membrane disruption.

Disruption of the outer bacterial membrane of P. aeruginosa was measured using the fluorescent probe 1-*N*-phenylnaphthylamine (NPN; Merck). Late-exponential-phase cultures of P. aeruginosa were washed twice and adjusted to an OD_600_ of 0.5 in 5 mM HEPES buffer. In the wells of a black microtiter plate (ThermoScientific), adjusted cultures were combined with NPN (final concentration, 10 μM) along with either no treatment (buffer only), 50 mg/L GA, 2 mg/L CST, 4 mg/L TOB, or 16 mg/L LL-37 (Merck), to a final volume of 200 μL. Agent concentrations were chosen based on EUCAST breakpoint concentrations (for CST and TOB) or previously published active concentrations (GA [18] and LL-37 [73]). Each experiment consisted of triplicate wells of each condition. Fluorescence was measured in a plate reader at excitation of 355 nm, emission at 460 nm every 30 s for 15 min. NPN uptake factor was calculated as follows: [(fluorescence of sample with NPN) − (fluorescence of sample without NPN)]/[(fluorescence of buffer with NPN) − (fluorescence of buffer without NPN)].

### Cytoplasmic membrane depolarization.

Depolarization of the cytoplasmic membranes of P. aeruginosa isolates was measured using the fluorescent probe DiSC_3_(5) (Thermo Scientific) (74). Late-exponential-phase cultures of P. aeruginosa were washed twice and adjusted to an OD_600_ of 0.05 in 5 mM HEPES–20mM glucose. DiSC_3_(5) was added to the bacterial cultures to a concentration of 1 μM and aliquoted to the wells of a black microtiter plate. The fluorescent signal of the dye was allowed to quench for 30 min in the dark followed by addition of equal volumes of buffer (no treatment), GA (for 50 mg/L), CST (2 mg/L), TOB (4 mg/L), or LL-37 (16 mg/L). Each experiment consisted of triplicate wells of each condition. Fluorescence was measured in a plate reader at excitation of 544 nm, emission at 620 nm every 30 s for 1 h.

### Bacterial membrane permeabilization.

Permeabilization of the bacterial membranes of P. aeruginosa was measured using the fluorescent dye PI per the manufacturer’s instructions (Merck). Late-exponential-phase cultures of P. aeruginosa were washed twice and adjusted to an OD_600_ of 0.5 in PBS. Propidium iodide was added to the bacterial cultures to a concentration of 1 μg/mL and treatments were added: no treatment, 50 mg/L GA, 2 mg/L CST, 4 mg/L TOB, or 16 mg/L LL-37. Cells were aliquoted to the wells of a black microtiter plate in 200-μL volumes. Each experiment consisted of triplicate wells of each condition. Fluorescence was measured in a plate reader at excitation at 544 nm, emission at 610 nm every 30 s for 1 h.

### Statistical analyses.

Bacterial growth curves were plotted by transformation of OD_600_ data using the natural log, of biological replicates, and displayed as median and 95% confidence intervals. The OD_600_ at 18 h and the AUC for biological replicates were compared by Kruskal-Wallis test with Dunn’s multiple comparison (to untreated), and adjusted *P* values are reported. Where the Kruskal-Wallis results were significant (*P* < 0.05), individual TOB concentrations were compared, with GA versus without GA, via the Mann-Whitney *t* test.

Synergy between the activities of glatiramer acetate and tobramycin was tested for each strain from colony counts, as previously described (38, 75). Briefly, the inhibition effects (in CFU per milliliter) caused by 50 mg/L GA (E_A_) and each TOB concentration tested (E_B_) were used to calculate the expected effect (E_Exp_) of combining both treatments, based on the equation E_EXP_ = E_A_ + E_B_ − (E_A_ × E_B_). The observed effect (E_OBS_) of the combined treatments was compared to the calculated E_Exp,_ whereby E_OBS_ greater than E_EXP_ indicates synergy, E_OBS_ equal to E_EXP_ indicates additivity or independence, and E_OBS_ less than E_EXP_ indicates antagonism. We report synergy when the above criteria were met, and the resulting values had nonoverlapping 95% confidence intervals.

Inhibition of each strain by TOB alone and by GA with TOB was calculated for each tobramycin concentration tested, as a percentage of the untreated cultures, using CFU results. Inhibition curves were generated for each P. aeruginosa strain using a nonlinear fit (inhibitor concentration versus response; three parameters). Tobramycin MIC_50_ and MIC_90_ values for viable bacteria were interpolated from the curves generated for TOB alone and GA plus TOB. This precise calculation also allowed statistical comparisons of the MIC_50_ and MIC_90_ results for tobramycin with and without GA, in a manner not possible with results of standard broth microdilution or disc diffusion testing. Median MIC_50_ and MIC_90_ values for all clinical strains were compared using Wilcoxon *t* tests.

Effects of different treatments on reference strains were compared for the median NPN uptake factor over 15 min, median DiSC_3_(5) release over 15 min, and median AUC of PI fluorescence intensity over 1 h by using the Kruskal-Wallis test with Dunn’s multiple comparisons (to untreated), and adjusted *P* values are reported. Clinical isolates were compared using the same membrane metrics for no treatment versus 50 mg/L GA with Wilcoxon signed-rank tests. All analyses were performed in GraphPad Prism (9.2). A *P* level of <0.05 was considered significant.
