# Supplementary material for: Synergistic Activity of Repurposed Peptide Drug Glatiramer Acetate with Tobramycin against Cystic Fibrosis Pseudomonas aeruginosa
Source: Microbiol Spectr. 2022 Jun 21;10(4):e00813-22. doi: 10.1128/spectrum.00813-22 (PMC9430792; doi:10.1128/spectrum.00813-22)
Supplement: Supplemental file 1 — Fig. S1 and S2; Tables S1 and S2. Download spectrum.00813-22-s0001.pdf, PDF file, 0.2 MB [file spectrum.00813-22-s0001.pdf]

Figure S1. Semilog plots of overnight growth of *P. aeruginosa* PA14 and PAK at 1mg/L TOB, with and without GA (natural logs of OD_{600}, median and 95% confidence intervals).

Figure S2. Inhibition Curves of *P. aeruginosa* strains PAO1, PA14 and PAK using Nonlinear Fit ([inhibitor] vs. response (three parameters)). Points show percent inhibition (of untreated control) (median and 95% confidence intervals). Tobramycin concentrations required to inhibit 50% (MIC_{50}) and 90% (MIC_{90}) of viable bacteria were interpolated from the curves generated for TOB-only and GA/TOB. Arrows indicate changes in the points where inhibition curves cross 50% and 90% when GA is present.

Supplementary Figure 1.

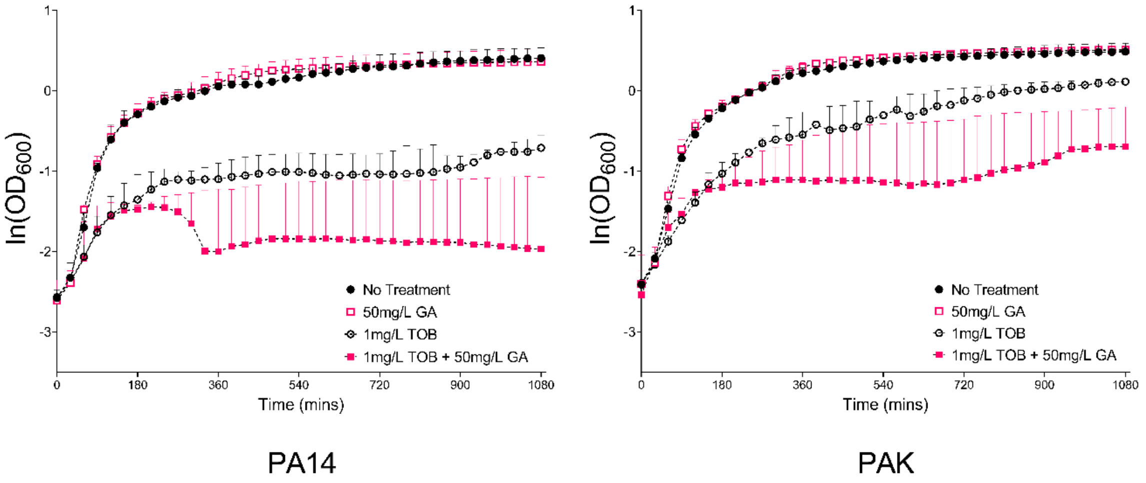

Supplementary Figure 2.

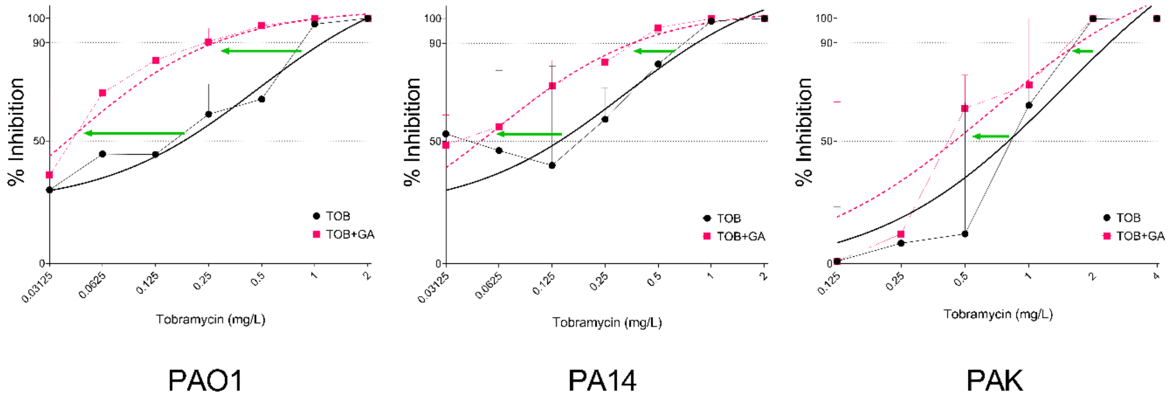

Supplementary Table 1. The MIC_{50} and MIC_{90} results for all *P. aeruginosa* strains tested in this study. Values and 95% confidence intervals interpolated for each strain from Inhibition Curves of CFU/mL results. Clinical strain colour codes match their representation in Figures 4 and 6.

	**MIC_{50}**			**MIC_{90}**		
	**TOB**	**GA/TOB**		**TOB**	**GA/TOB**	
**PAO1**	**0.23**<br>[0.10 - 0.63]	**0.05**<br>[0.01 - 0.11]		**1.18**<br>[0.45 -]	**0.25**<br>[0.09 - 0.74]	
**PA14**	**0.12**<br>[- 0.26]	**0.04**<br>[0.08 -]		**0.86**<br>[0.50 -]	**0.44**<br>[0.24 -]	
**PAK**	**0.86**<br>[0.51 - 1.48]	**0.45**<br>[0.23 - 0.86]		**2.49**<br>[1.47 -]	**1.71**<br>[0.83 -]	
**RBH750**	**0.23**<br>[0.15 - 0.35]	**0.08**<br>[0.04 - 0.13]		**1.18**<br>[0.81 -]	**0.69**<br>[0.44 – 1.41]	
**RBH899**	**0.26**<br>[0.12 - 0.56]	**0.22**<br>[0.10 – 0.46]		**1.15**<br>[0.55 -]	**0.99**<br>[0.50 -]	
**RBH550**	**0.51**<br>[0.33 – 0.78]	**0.34**<br>[0.22 - 0.54]		**1.30**<br>[0.81 –]	**1.06**<br>[0.60 -]	
**RBH294**	**0.53**<br>[0.30 – 0.95]	**0.55**<br>[0.32 – 0.97]		**2.33**<br>[1.37 –]	**2.21**<br>[1.32 –]	
**RBH982**	**0.70**<br>[0.35 – 1.38]	**0.15**<br>[– 0.36]		**2.60**<br>[1.30 –]	**1.21**<br>[0.53 –]	
**RBH422**	**2.21**<br>[1.50 – 3.31]	**0.69**<br>[0.58 – 0.83]		**7.00**<br>[4.39 –]	**2.8**<br>[2.00 – 3.97]	
**RBH519**	**1.69**<br>[– 4.67]	**0.62**<br>[– 0.92]		**12.76**<br>[3.11 –]	**2.10**<br>[1.00 –]	
**RBH461**	**3.90**<br>[– 7.69]	**2.23**<br>[– 4.13]		**17.16**<br>[9.11 –]	**11.12**<br>[5.43 –]	
**RBH490**	**5.57**<br>[3.67 – 8.18]	**1.18**<br>[0.76 – 1.98]		**12.23**<br>[8.49 –]	**4.50**<br>[2.27 –]	
**RBH065**	**8.97**<br>[5.17 – 15.14]	**3.94**<br>[– 8.05]		**26.50**<br>[15.78 –]	**15.03**<br>[6.98 –]	

**RBH072**	**211.19**<br>[125.06 – 333.84]	**62.46**<br>[49.21 – 82.47]		**438.32**<br>[288.05 –]	**251.49**<br>[179.36 –]	●
**Clinical Strain Median**	**1.69**<br>[0.26 – 8.97]	**0.62**<br>[0.15 – 3.94]		**7.00**<br>[1.18 – 15.03]	**2.21**<br>[0.99– 15.03]	

Supplementary Table 2. *P. aeruginosa* strains which showed synergy for the combination of glatiramer acetate and tobramycin and the tobramycin concentration at which that synergy occurred.

Strain	TOB Concentration
**PAO1**	0.25mg/L
	0.5mg/L
**PA14**	1mg/L
**PAK**	2mg/L
**RBH750**	2mg/L
**RBH982**	2mg/L
**RBH422**	4mg/L
**RBH519**	1mg/L
	4mg/L
**RBH490**	2mg/L
	4mg/L
	8mg/L
**RBH072**	128mg/L
